# Sphingolipidomics of Bovine Pink Eye: A Pilot Study

**DOI:** 10.3390/vetsci9080388

**Published:** 2022-07-28

**Authors:** Paul L. Wood, Lynda M. J. Miller

**Affiliations:** 1 Metabolomics Unit, College of Veterinary Medicine, Lincoln Memorial University, 6965 Cumberland Gap Pkwy, Harrogate, TN 37752, USA; 2 College of Veterinary Medicine, Lincoln Memorial University, 6965 Cumberland Gap Pkwy, Harrogate, TN 37752, USA; linda.miller@lmunet.edu

**Keywords:** bovine pink eye, Infectious Bovine Keratoconjunctivosous (IBK), sphingolipids

## Abstract

**Simple Summary:**

The liquid tear film, which protects the eye from the environment, is a dynamic fluid containing a large number of complex lipids. Disruptions of these lipids by infections can result in damage to the eye and ultimately blindness. In this study we characterized various lipid subfamilies present in the tear film of the eye and the effect of pink eye infections in cattle. Our findings demonstrate that the pink eye infections dramatically decrease the levels of lipids in the tear film covering the eye and suggest that this is a major factor in the development of blindness in infected cattle.

**Abstract:**

Sphingolipids are essential structural components of tear film that protect the surface of the eye from dehydration. A detailed analysis of the effects of pink eye infections on the sphingolipidome in cattle has not previously been undertaken. We recently published a new assay utilizing high-resolution mass spectrometric monitoring of the chloride adducts of sphingolipids that provides enhanced sensitivity and specificity. Utilizing this assay, we monitored decreases in the levels of tear film ceramides with short-chain fatty acids, hydroxy-ceramides, phytoceramides, and hydroxy-phytoceramides. Dihydroceramide levels were unaltered and increased levels of ceramides with long-chain fatty acids (24:0 and 24:1) were monitored in cattle with pink eye. The data from this pilot study (n = 8 controls and 8 pink eye) demonstrate a major disruption of the lipid tear film layer in pink eye disease, that can result in severe eye irritation and damage.

## 1. Introduction

The tear film covering of the eye is structurally complex with 2 major layers: (1) an aqueous-mucin layer over the surface epithelium of the eye, which is secreted by surface epithelial cells and goblet cells and (2) an outer lipid layer composed of polar lipids which are secreted by the Meibomian glands, serving as the interface between the aqueous layer and the air [1,2,3].

The tear film is a dynamic structure protecting the eye surface from dehydration, physical abrasion from blinking, and provides immune protection against pollutants and pathogens. Tear film infections, like bovine pink eye, lead to a disruption of tear film stability by affecting the lipid components of the outer lipid layer and by inducing cytoprotective biochemical pathways [1,2,3].

Lipidomics analyses of the tear film have revealed that the lipid layer is extremely complex being composed of glycerophospholipids (diacyl and plasmalogens), lysophosphatidic acids, cyclic phosphatidic acids, sphingomyelins, fatty acids, fatty acyl esters of hydroxy-fatty acids (FAHFA), triglycerides, cholesterol esters, and wax esters [3,4,5,6]. In our previous lipidomics analyses of bovine pink eye [6], we monitored induction of anti-inflammatory lipids, including cyclic phosphatidic acids and resolvins. We also monitored increases in the levels of structural lipid components including FAHFAs (also termed o-acyl-hydroxy-fatty acids [6]), ethanolamine plasmalogens, and sphingomyelins.

However, our direct infusion assay was limited in sensitivity for a number of sphingolipids. Sphingolipids are important structural components of membranes and tear film, with hydroxy sphingolipids representing stabilizers due to their increased hydrogen bonding capabilities. Alterations in these hydroxy lipids can disrupt the tear film layer resulting in dehydration and abrasion to the eye. With these issues in mind, we undertook a more in-depth high-resolution mass spectrometric analysis of the tear film sphingolipidome utilizing a new assay of the chloride adducts of these lipids, which provides improved sensitivity and specificity [7,8]. 

## 2. Materials and Methods

### 2.1. Clinical Samples

A herd of approximately 1200 Black Angus cattle of various ages (0–15 years) were used in this study. The cattle were visually evaluated in groups of 70–100 at least every 30 days for active Infectious Bovine Keratoconjunctivitis (IBK). The prevalence rate of pink eye each spring in this herd is 25%. When active Stage 3 IBK infection was visually noted, samples were collected for lipidomics analyses. A picture of the IBK affected eye(s) was captured and a sample of ocular (tear) fluid was collected by placing 2 cotton tipped swabs in the lower eyelid. The cotton tipped swabs were placed in 1 mL of methanol and transported to the laboratory for analyses. Following sample collection, the affected animals were treated with tulathromycin or gamithromycin and an eye patch was applied to the severe cases. The pictures were evaluated to determine the Stage of the pinkeye infection as described by W.D. Whittier et al. (Supplementary Clinical Data). The veterinarian collecting the samples also staged the disease in each animal. 

### 2.2. Lipidomics

To the cotton swabs in 1 mL of methanol were added 1 mL of water and 2 mL of methyl-tert-butyl ether. The tubes were next vigorously shaken at room temperature for 30 min. prior to centrifugation at 4000× *g* for 30 min. at room temp. The upper organic layer was isolated and dried by centrifugal vacuum evaporation. This protocol is identical to our previous publications [7,8] except for a modification in the composition of the infusion solution: 2-propanol: methanol: chloroform: H_2_O (160:80:80:1) with the water containing 15 mg of NH_4_Cl.

Direct infusion lipidomics utilized high-resolution data acquisition (≤1 ppm mass error), with an orbitrap mass spectrometer (Thermo Q Exactive). In negative ion ESI, the chloride adducts [7,8] of ceramides, dihydroceramides, hydroxy-ceramides, phytoceramides (Cer X-O3), and hydroxy-phytoceramides (Cer X-O4 and Cer X-O5) were reliably monitored (Supplementary Table S1). Hexosyl-ceramides, lactosyl ceramides, acyl-ceramides, deoxyceramides, and ceramide-phosphoethanolamines were not reliably detected. Between injections, the transfer line was washed with successive 500 µL washes of methanol and hexane/ethyl acetate/chloroform/H_2_O (3:2:1:0.1). 

Validation of infection was also investigated with high-resolution mass spectrometric analysis of bacterial membrane trehalose monohydroxylcorynomycolates (hTMCM; [9]) in the tear film extracts.

Validation of the phytoceramides and hydroxyceramides as t18:0/X was achieved via tandem mass analyses with positive ESI. In all cases the double dehydrated cation of t18:0 (282.2792) was monitored as a product ion with <2 ppm mass error. For MS^2^ analyses, an isolation window of 0.4 amu and collision energies of 10, 20, and 30 NCE were used.

### 2.3. Statistical Analysis

Since the samples were absorbed onto cotton swabs for extraction, we had no index of the volume collected. Therefor standardization of the data, based on tear volume, was not possible. In this regard, we expressed the peak intensity of individual sphingolipids as a ratio to the peak intensity of endogenous Cer d18:1/16:0 in each tear film sample. This is a valid comparator since we have previously shown that Cer d18:1/16:0 levels are unaltered in pink eye infections [4]. The data presented in graphs are from a pilot study with 8 controls and 8 cattle with pink eye. A Student’s *t*-test (Microsoft Excel), assuming equal or unequal variances, was used to determine significant differences in relative levels of metabolites between pink eye animals and controls, subsequent to an F-test (Microsoft Excel) to determine if the variances between groups were statistically different.

## 3. Results

### 3.1. Clinical Samples

For the lipidomics analyses, 8 controls with no clinical signs of eye infections were selected for our pilot study along with 8 cattle with stage 3 pink eye infections.

### 3.2. Ceramides

**Figure 1:**
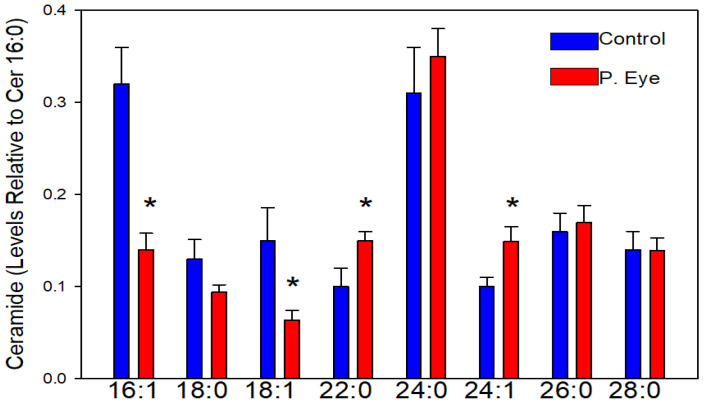
Tear film levels of ceramides (Cer d18:1/x) in controls and cows with pink eye (P. Eye). The peak intensities of individual ceramides are expressed as a ratio of the peak intensity of ceramide d18:1/16:0. *, significant *t*-test statistics: 16:1 (0.013); 18:1 (0.021); 22:0 (0.027); 24:1 (0.039).

The chloride adducts of 24 ceramides were reliably monitored. In Figure 1, we report the ceramides with a signal-to-noise ratio >5. The levels of ceramides with 18:0, 24:0, 26:0, and 28:0 fatty acid substituents were unaltered in the tear film of cattle infected with pink eye (Figure 1). In contrast, Cer d18:1/16:1 and Cer d18:1/18:1 were decreased, while Cer d18:1/22:0 and Cer d18:1/24:1 were augmented (Figure 1). Tear film ceramides have been reported previously to be decreased with meibomian gland dysfunction and parasitic infections [10,11,12,13]. This is important in that these sphingolipids are integral in maintaining the structural stability of the tear film which protects the ocular surface from abrasion, irritants and infections [10,11,12,13,14,15].

### 3.3. Hydroxy-Ceramides

**Figure 2:**
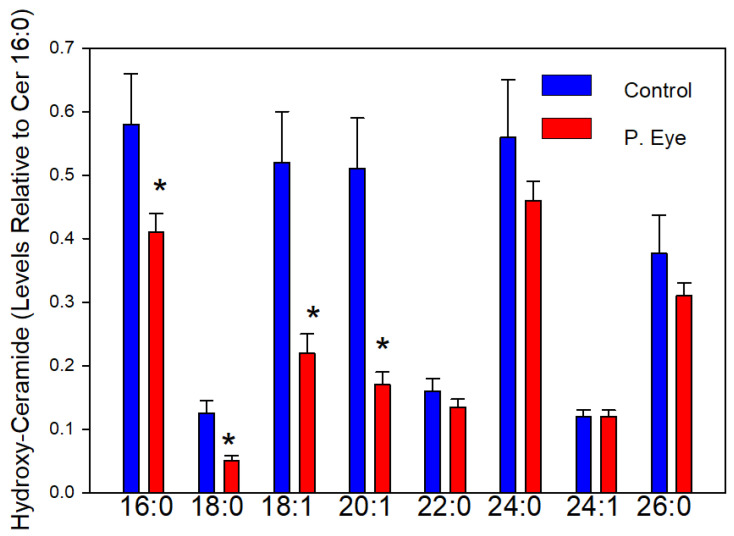
Tear film levels of hydroxy-ceramides (Hydroxy-Cer d18:1/x) in controls and cows with pink eye (P. Eye). The peak intensities of individual hydroxy-ceramides are expressed as a ratio of the peak intensity of ceramide d18:1/16:0. *, significant *t*-test statistics: 16:0 (0.023); 18:0 (0.014); 18:1 (0.020); 20:1 (0.0083).

The chloride adducts of 19 hydroxy-ceramides were reliably monitored. In Figure 2, we report the hydroxy-ceramides with a signal-to-noise ratio >5. Hydroxyceramides with short-chain 2-hydroxy fatty acid substituents were consistently decreased in the tear film of infected cattle (Figure 2). Control levels of hydroxy-ceramides were approximately 2 times the control levels of ceramides in bovine tear film (Figure 1). These lipids can be formed de novo by addition of a hydroxy fatty acid rather than a fatty acid or by lipid remodeling involving deacylation followed by reacylation with a hydroxy fatty acid. This is the first report, to-date, of hydroxy-ceramides in tear film. The greater hydrogen bonding capabilities of hydroxy-ceramides increases their ability to interface between the aqueous and lipid layers of tear film [16,17,18,19,20,21,22].

### 3.4. Phytoceramides (Cer X-O3)

**Figure 3:**
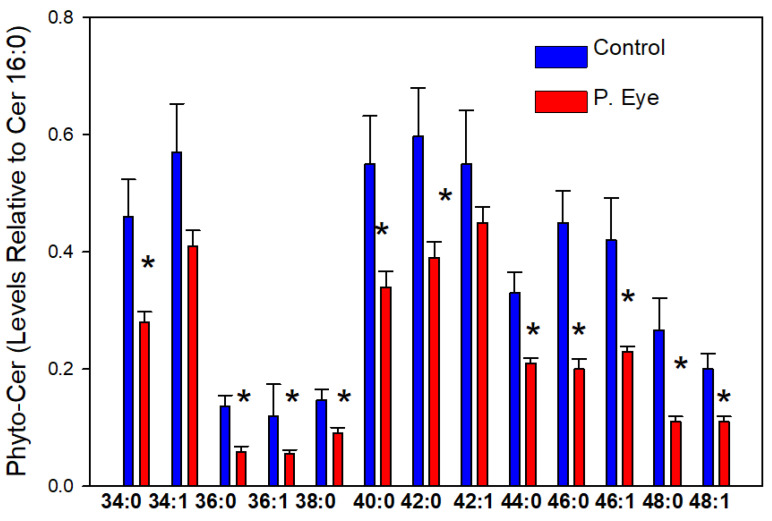
Tear film levels of phytoceramides (Phyto-Cer) in controls and cows with pink eye (P. Eye). The peak intensities of individual phytoceramides are expressed as a ratio of the peak intensity of ceramide d18:1/16:0. *, significant *t*-test statistics: 34:0-O3 (0.024), 36:0-O3 (0.016), 36:1-O3 (0.01), 38:0-O3 (0.011), 40:0-O3 (0.040), 42:0-O3 (0.043), 44:0-O3 (0.036), 46:0-O3 (0.012), 46:1-O3 (0.034), 48:0-O3 (0.0086), and 48:1-O3 (0.026).

The chloride adducts of 25 phytoceramides were reliably monitored. In Figure 3 we report the phytoceramides with a signal-to-noise ratio >5. The levels of tear film phytoceramides were consistently decreased in pink eye infections (Figure 3). Phytoceramides are generated by the addition of an additional hydroxy group (hence Cer X-O3) to the sphinganine base of a dihydroceramide and by direct synthesis from phytosphinganine. This is the first report, to-date, of phytoceramides in tear film. The greater hydrogen bonding capabilities of phytoceramides increases their ability to interface between the aqueous and lipid layers of tear film [16,17,18,19,20,21,22].

### 3.5. Hydroxy-Phytoceramides (Cer X-O4)

**Figure 4:**
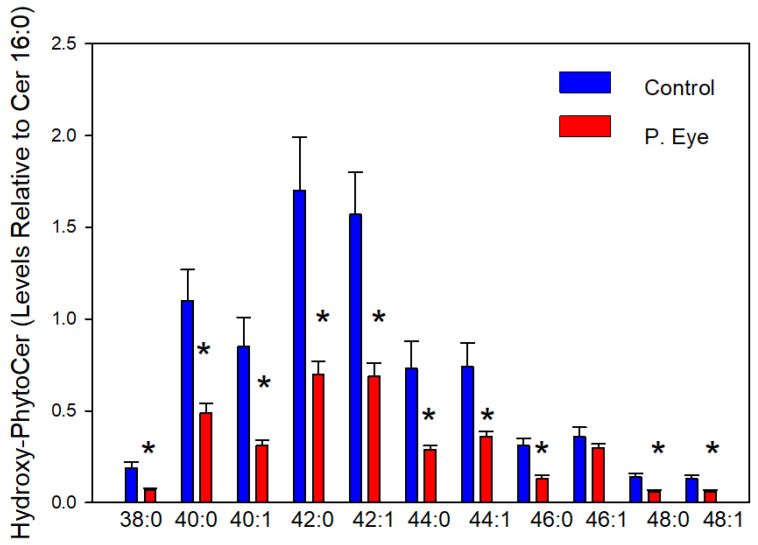
Tear film levels of hydroxy-phytoceramides (CerX-O4) in controls and cows with pink eye (P. Eye). The peak intensities of individual hydroxy-phytoceramides are expressed as a ratio of the peak intensity of ceramide d18:1/16:0. *, significant *t*-test statistics: 38:0-O4 (0.0064), 40:0-O4 (0.0056), 40:1-O4 (0.0070), 42:0-O4 (0.0075), 42:1-O4 (0.0048), 44:0-O4 (0.013), 44:1-O4 (0.015), 46:0-O4 (0.0030), 48:0-O4 (0.10), and 48:1-O4 (0.018).

The chloride adducts of 22 hydroxy-phytoceramides (Cer-X-O4) were reliably monitored. In Figure 4 we report the Cer X-O4s with a signal-to-noise ratio >5. The levels of tear film Cer X-O4 lipids were all decreased in infected cattle (Figure 4). In this class of lipids, a 2-hydroxy fatty replaces the fatty acid substituent. This can take place via de novo biosynthesis or via lipid remodeling. This is the first report, to-date, of Cer X-O4s in tear film. The greater hydrogen bonding capabilities of these sphingolipids increases their ability to interface between the aqueous and lipid layers of tear film [16,17,18,19,20,21,22].

### 3.6. Hydroxy-Phytoceramides (Cer X-O5)

**Figure 5:**
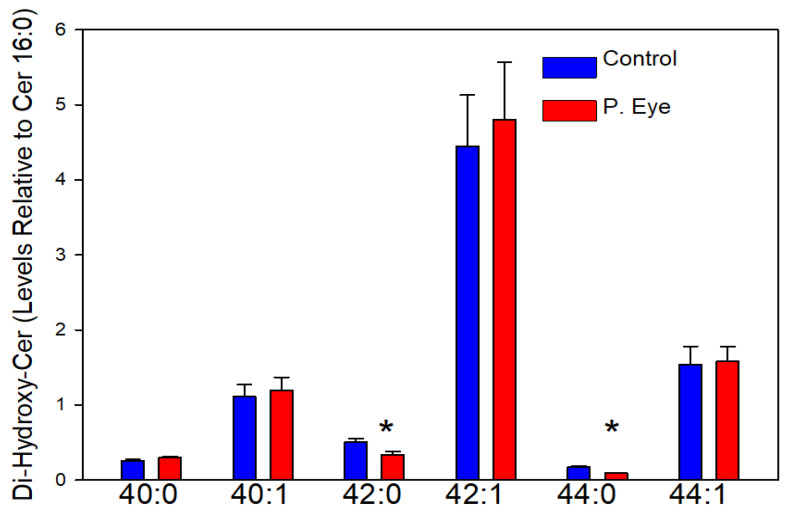
Tear film levels of di-hydroxy-phytoceramides (CerX-O5) in controls and cows with pink eye (P. Eye). The peak intensities of individual di-hydroxy-phytoceramides are expressed as a ratio of the peak intensity of ceramide d18:1/16:0. *, significant *t*-test statistics: 42:0-O5 (0.021) and 44:0-O5 (0.0090).

The chloride adducts of 11 hydroxy-phytoceramides (Cer X-O5) were reliably monitored. In Figure 5 we report the Cer X-05s with a signal-to-noise ratio >5. The diversity of tear film Cer X-O5 lipids (Figure 5) was dramatically less than our observations with Cer X-O3 (Figure 3) and Cer X-O4 (Figure 4) phytoceramides. In this class of lipids, replacement of the fatty acid with a dihydroxy fatty acid in the biosynthesis of phytoceramides occurs. For these lipids, only Cer 42:0-O5 and Cer 44:0-O5 were decreased (Figure 5). This is the first report, to-date, of Cer X-O5s in tear film. The greater hydrogen bonding capabilities of these sphingolipids increases their ability to interface between the aqueous and lipid layers of tear film [16,17,18,19,20,21,22].

### 3.7. Dihdroceramides

The chloride adducts of 8 dihydroceramides were reliably monitored. Of dihydroceramides with a signal-to-noise ratio >5, the tear film levels of dihydroceramides 16:0, 18:0, 20:0, 22:0 and 24:0 were unaltered with pink eye. Dihydroceramides are the direct precursors of both ceramides and phytoceramides.

### 3.8. Biomarkers of Ocular Infection

The chloride adducts of 3 bacterial membrane trehalose monohydroxylcorynomyclates [9] were reliably monitored in the tear film of cattle with pink eye and were absent from control cows (Table 1). These complex bacterial membrane saccharolipids are composed of a long-chain hydroxy fatty acid with an acyl linkage to the disaccharide, trehalose. The levels of trehalose monohydroxylcorynomyclates were low but detectable with a signal to noise of greater than 5. The monitored trehalose monohydroxylcorynomyclates are precursors of mycolic acids, virulence factors of bacteria [9]. We presume that in our study they are biomarkers of *Moraxella bovis*, the most frequent bacteria responsible for most pink eye infections.

## 4. Discussion

Previous studies, like this study, have demonstrated a diversity of ceramides in tear film [10,11,12,13], with decrements of these lipids in dry eye [11,12] and in eye infections with the ectoparasite *desmodex* [13]. These published data and our observations suggest that there are significant decrements in tear film ceramides with infection and meibomian gland dysfunction. Ceramides are polar lipids that interface between the outer non-polar lipids and the inner polar lipid layer that is associated with the inner aqueous phase, acting to stabilize the tear film [14,15]. It has been hypothesized that the hydrogen bonding properties of the head groups of these lipids contribute to maintaining the tear film structure [14,15].

**Figure 6:**
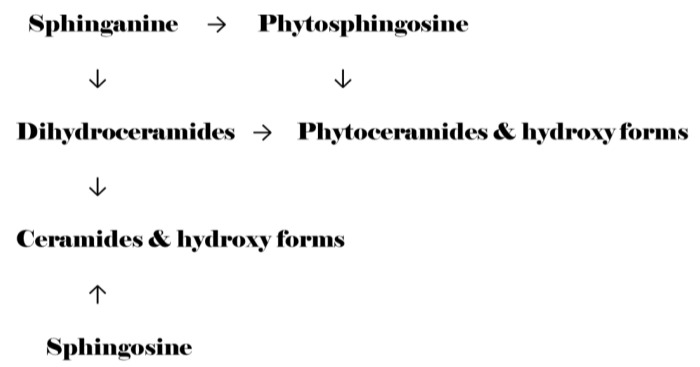
Schematic of sphingolipid metabolism. Hydroxylation of free sphingolipid bases is catalyzed by monooxygenases (SUR2) and of sphingolipid bases in dihydroceramides via hydroxylases (DES2). The introduction of hydroxylation at position 2 of the fatty acid substituents of ceramides occurs via de novo synthesis with a hydroxy fatty acid (CERS, ceramide synthase) and via lipid modification by ceramide fatty acyl 2-hydroxylase (FA2H; phytoceramide → 2-hydroxy-phytoceramide).

While previous investigators have studied ceramides, hexosylceramides and sphingomyelins in tear film, they have not reported on the multiple hydroxy forms of sphingolipids [3,4,5]. Of particular interest are hydroxy-sphingolipids since these lipids are capable of enhanced hydrogen bonding [16]. Two major hydroxylation patterns are present in most biological systems. Namely hydroxylation of the sphingolipid base or of the fatty acid substituent. Sphinganine monooxygenase [17], (EC 1.14.18.5) introduces a hydroxy group into the sphingolipid base of sphinganine to generate phytosphingosine and into the sphingolipid base of dihydroceramides to generate phytosphingosines (Figure 6). Phytosphingosines can also be formed via direct hydroxylation of the sphingolipid base of dihydroceramides, catalyzed by dihydroceramide hydroxylase [18], (DES2). De novo generation of 2-hydroxy-phytoceramides is catalyzed by a large family of ceramide synthases [19], (CERS; 2-hydroxy fatty acid CoA + sphingolipid base → hydroxy-phytoceramide) and by direct hydroxylation of a phytoceramide via ceramide fatty acyl 2-hydroxylase [20,21,22], (FA2H). CERS can also generate 2-hydroxyceramides by addition of a 2-hydroxy fatty acid CoA to sphingosine (Figure 6). Conversion of sphingosine to ceramides and to hydroxyceramides is also catalyzed by the CERS family of enzymes (Figure 6). Omega-hydroxyceramides have also been reported in the literature [23]; however, these lipids are covalently bound to cellular membrane proteins via the ω-hydroxy group and are not extracted without prior hydrolysis steps.

Since we found no alterations in the levels of tear film dihydroceramides but decrements in ceramides, hydroxyceramides, phytoceramides and hydroxy-phytoceramides, the SURS, CERS, DES2, and FA2H enzyme systems must be dysfunctional in bovine pink eye infections. These deficiencies will ultimately result in increased shear forces on the surface of the eye and increased risk of dehydration. In addition, these lipids may function in an antimicrobial role to protect the eye [24,25], such that their decrements may accelerate microbial proliferation.

The ocular tear film is a 3 to 10 µm layer with an outer non-polar lipid layer and an inner polar lipid layer that interfaces with the aqueous layer covering the eye surface [14,15,16,17,18]. Tear film flows continuously to clear environmental debris and pathogens [20,21,22,23,24,25]. The lipid layers slow evaporation from the aqueous layer thereby protecting the eye surface from abrasion, pathogens, and inflammation. Hence, the structural integrity of tear film lipids is essential to maintain ocular function. The interphase of the aqueous and polar lipid layers is made possible by a number of amphiphilic lipids [2,3,6] and by polar lipids with hydroxy-fatty acid substituents. The hydroxy fatty acids provide enhanced hydrogen bonding with the aqueous film. Our study is the first to demonstrate that hydroxyceramides and hydroxyphytoceramides are present in tear film and are strong candidates for stabilizing the lipid-aqueous interface in tear film. Furthermore, disruption of these lipids in pink eye infections will be a major factor in the decline of ocular function in infected cattle.

## 5. Limitations and Future Directions

This was a pilot study with a small sample size therefore requiring a validation study in a larger population of cattle. We are currently collecting a larger sample set to address this issue, to monitor the time course of lipid changes, and to monitor lipid responses to antibiotic treatment.

## 6. Conclusions

This is the first report demonstrating the presence of hydroxyceramides, phytoceramides, and hydroxy-phytoceramides in tear film. As a result of the interconnected pathways in the generation and metabolism of hydroxy sphingolipids, a detailed pathway analysis cannot be generated until a number of the key enzymes have been quantitated. The decrements in a number of these hydroxy sphingolipids results in eye dehydration and possibly decreased antimicrobial protection. 

## Figures and Tables

**Table 1:** The chloride adducts of bacterial membrane trehalose monohydroxylcorynomyclates monitored in the tear film of cattle with pink eye.

Lipid	Exact Mass	[M+Cl]^−^	Level Relative to Cer d18:1/16:0	ppm
hTMCM 28:2	760.4973	795.4671	0.0051 ± 0.0015	0.44
hTMCN 33:3	828.5599	863.5297	0.010 ± 0.0015	1.5
hTMCM 34:3	842.5755	877.5454	0.0070 ± 0.0023	1.3

## Data Availability

Raw data files are available to qualified investigators.

## Supplementary Materials

The following supporting information can be downloaded at: https://www.mdpi.com/article/10.3390/vetsci9080388/s1, Table S1: Lipid masses.
